# ERK MAPK signaling pathway inhibition as a potential target to prevent autophagy alterations in Spinal Muscular Atrophy motoneurons

**DOI:** 10.1038/s41420-023-01409-x

**Published:** 2023-04-05

**Authors:** Alba Sansa, Maria P. Miralles, Maria Beltran, Ferran Celma-Nos, Jordi Calderó, Ana Garcera, Rosa M. Soler

**Affiliations:** 1 Neuronal Signaling Unit, Experimental Medicine Department, Universitat de Lleida-IRBLleida, Rovira Roure, 80, 25198 Lleida, Spain; 2 Patologia Neuromuscular Experimental, Experimental Medicine Department, Universitat de Lleida-IRBLleida, Rovira Roure, 80, 25198 Lleida, Spain

**Keywords:** Neurodegeneration, Cell signalling

## Abstract

Spinal Muscular Atrophy (SMA) is a severe genetic neuromuscular disorder that occurs in childhood and is caused by misexpression of the survival motor neuron (SMN) protein. SMN reduction induces spinal cord motoneuron (MN) degeneration, which leads to progressive muscular atrophy and weakness. The link between SMN deficiency and the molecular mechanisms altered in SMA cells remains unclear. Autophagy, deregulation of intracellular survival pathways and ERK hyperphosphorylation may contribute to SMN-reduced MNs collapse, offering a useful strategy to develop new therapies to prevent neurodegeneration in SMA. Using SMA MN in vitro models, the effect of pharmacological inhibition of PI3K/Akt and ERK MAPK pathways on SMN and autophagy markers modulation was studied by western blot analysis and RT-qPCR. Experiments involved primary cultures of mouse SMA spinal cord MNs and differentiated SMA human MNs derived from induced pluripotent stem cells (iPSCs). Inhibition of the PI3K/Akt and the ERK MAPK pathways reduced SMN protein and mRNA levels. Importantly, mTOR phosphorylation, p62, and LC3-II autophagy markers protein level were decreased after ERK MAPK pharmacological inhibition. Furthermore, the intracellular calcium chelator BAPTA prevented ERK hyperphosphorylation in SMA cells. Our results propose a link between intracellular calcium, signaling pathways, and autophagy in SMA MNs, suggesting that ERK hyperphosphorylation may contribute to autophagy deregulation in SMN-reduced MNs.

## Introduction

Spinal Muscular Atrophy (SMA) is a neuromuscular disease characterized by degeneration of alpha motoneurons (MNs) located in the ventral horn of the spinal cord [1], leading to muscle wasting and paralysis [2, 3]. This disease affects 1 in 6000 to 10,000 live births and is the most common cause of infant death of genetic origin [4]. SMA is caused by point mutations and/or homozygous deletion of the *Survival motor neuron 1 (SMN1)* gene, located at the telomeric region of the chromosome 5q3, resulting in deficient levels of SMN protein. It is well known that SMN deficiency provokes cellular degeneration, although the underlying molecular mechanisms are not fully understood [5, 6].

MN development and physiology relies on a continuous and regulated trophic support induced by neurotrophic factors (NTFs) coming from innervated tissues, afferent neurons and glial cells [7, 8]. NTFs limit neuronal cell death during development by regulating cell survival. They trigger several cascades of survival signaling pathways through the activation of membrane receptors [9]. Two of these pathways, PI3K/Akt and ERK MAPK, are crucial for the survival and maintenance of a wide range of neuronal populations. Akt activation can lead to the phosphorylation of RXRXX(S/T) sequences of transcription factors, controlling apoptosis or pro-survival molecules [10]. For instance, PI3K/Akt activation by NTFs mediates in vitro MN survival, whereas their inhibition causes apoptotic cell death [11, 12]. ERK MAPK pathway activation can lead to the nuclear translocation of phosphorylated ERK protein, activating transcription factors such as CREB, c-Myc, Elk1 and c-Jun, and controlling cell cycle, migration, differentiation, survival and apoptosis processes [13]. Both PI3K/Akt and ERK MAPK pathways have been described to be compromised in SMN-deficient cells [14–16]. In SMA cells, Akt phosphorylation is reduced and ERK phosphorylation is increased; in cultured mouse MNs, PI3K inhibition reduced Smn protein and mRNA level [14]. In contrast, ERK was found to be constitutively overactivated in mouse SMA spinal cord [17] and in human differentiated SMA MNs [14].

While the canonical view of ERK emphasizes its anti-apoptotic properties, its activity has been linked to different modes of cell death [18]. Among other functions, ERK participates in the regulation of mTOR1 protein [19]. Several upstream signals, including ERK, converge on the phosphorylation and inhibition of Tuberous Sclerosis Complex (TSC) to activate mTORC1 [20, 21]. mTOR functions as the catalytic subunit of two individual complexes, known as mTORC1 and mTORC2. Specifically, mTORC1 inhibits the Unc-51 like autophagy activating kinase 1 (ULK1), an essential protein for the initiation of the autophagy process [22].

Our objectives are to further analyze the contribution of PI3K/Akt and ERK MAPK pathways on SMN protein regulation in MNs, and to investigate whether these intracellular pathways participate in autophagy deregulation in SMA cells. Results provided evidence indicating that inhibition of ERK or Akt signaling pathways decreased SMN at both protein and mRNA levels in human differentiated MNs from induced pluripotent stem cells (IPSCs). We also found that increased ERK phosphorylation observed in SMN-reduced cells may contribute to the autophagy alterations in SMA MNs. Pharmacological inhibition of the ERK pathway reduced mTOR phosphorylation, p62, and LC3-II protein levels in SMA differentiated MNs. Importantly, treatment with the intracellular calcium chelator BAPTA prevented ERK overphosphorylation in SMA cells, suggesting a link between calcium, signaling pathways, and autophagy in SMA cells. Our data provide new insights on the molecular mechanisms leading to MN degeneration in SMA MNs, and identify new potential targets for SMA therapeutic strategies.

## Results

### PI3K/Akt intracellular pathway inhibition decreases SMN in human differentiated MNs

**Fig. 1:**
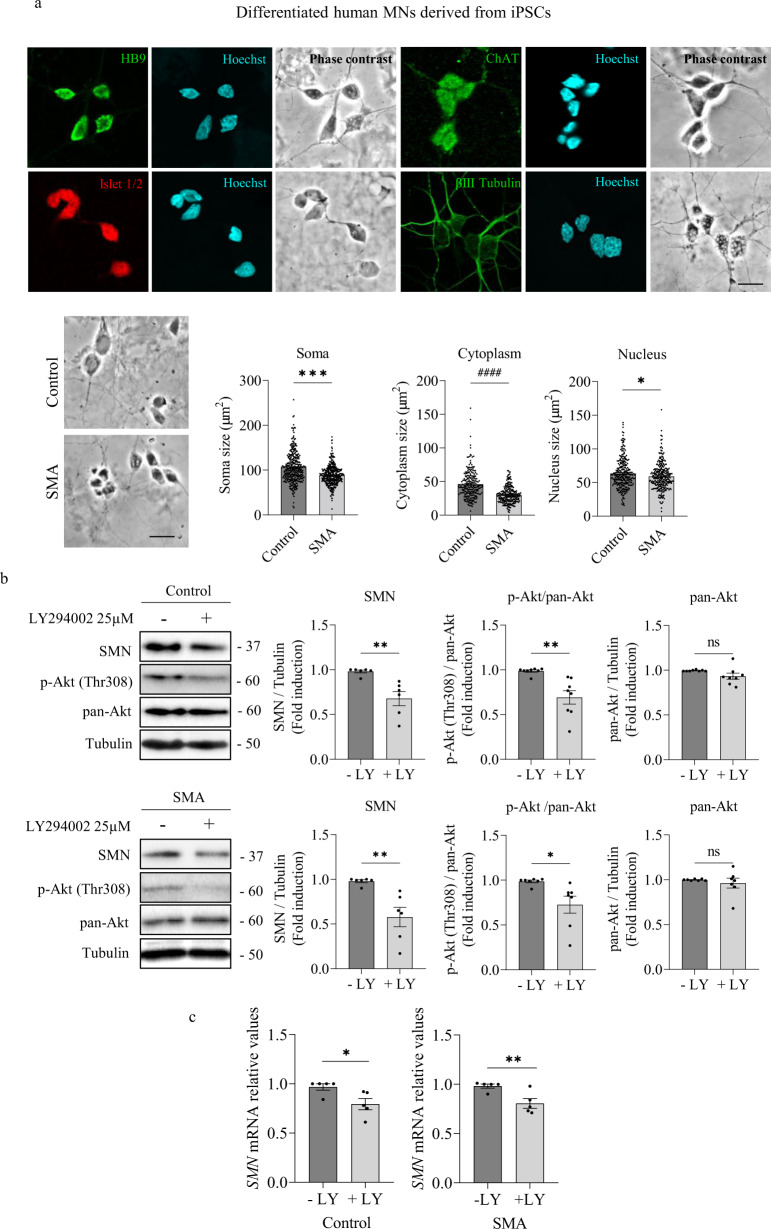
PI3K inhibition reduces SMN level in human differentiated MNs. **a** Representative phase contrast and immunofluorescence images of 7-day differentiated human MNs, showing the MN markers HB9 and Islet 1/2 (green and red, respectively); ChAT and beta-III-tubulin (green, upwards and downwards, respectively). Hoechst (blue) staining was used to identify MN nuclei. Scale bar 15μm. Graphs represent the mean size (expressed in μm^2^) of cell soma, cytoplasm, and nuclei of differentiated Control and SMA human MNs (representative images of Control and SMA, left panels), from the quantification of 3 independent iPSCs differentiation cycles ± SEM; each point corresponds to a single cell. Symbols indicate significant differences using Student *t* test (*p < 0.05; ***p < 0.0001) or Mann-Whitney test (^####^*p* < 0.0001). **b** Seven-day differentiated Control (left) and SMA (right) human MNs were washed and treated or not with 25 μM LY294002. At 24 h, protein extracts were obtained and submitted to western blot analysis using anti-phospho-Akt (Thr308) (p-Akt) antibody and anti-SMN antibody. Membranes were stripped and reprobed with anti-Akt (pan-Akt) antibody or with anti-α-tubulin antibody. Graphs represent the expression of SMN vs α-tubulin or p-Akt vs pan-Akt or pan-Akt vs α-tubulin, corresponding to the quantification of 6 independent iPSCs differentiation cycles ± SEM. Asterisks indicate differences using Student *t* test (*p < 0.05; **p < 0.005). **c** Total RNA was extracted from 25 μM LY294002-treated (+LY) or non-treated (-LY) 7-day differentiated Control and SMA human MNs. RNA was reverse transcribed to cDNA and *SMN* was amplified through RT-qPCR. *GAPDH* was used as control. Graph values are the mean of *SMN* gene expression from 3 independent differentiation cycles ± SEM. Asterisks indicate significant differences using Student *t* test (*p < 0.05; **p < 0.01).

To explore the role of PI3K/Akt pathway on SMN regulation, human iPSCs from an SMA patient and a non-affected control (Control) (from Coriell Institute, see Materials and Methods) were differentiated to MNs (Fig. 1a). After 7 days, Islet 1/2, HB9, and ChAT MN markers and β-III-tubulin were expressed by these cells, suggesting that iPSCs were successfully differentiated.

We observed that SMA MNs were significantly smaller than Control cells (Soma: Control 106.3 ± 1.82, SMA 88.74 ± 1.16, *p* < 0.0001; Cytoplasm: Control 45.91 ± 1.35, SMA 29.94 ± 0.77, *p* < 0.0001; Nucleus: Control 63.37 ± 1.34, SMA 59.07 ± 1.15, *p* = 0.015) (Fig. 1a). Seven days after differentiation, Control and SMA cultures were treated with 25 µM of the PI3K inhibitor LY294002 (Sigma). Twenty-four hours later, protein extracts were obtained and western blot analysis of phospho-Akt (Thr308) (p-Akt), Akt (pan-Akt), and SMN protein level was performed. As previously described [14], total Akt protein level (0.61 ± 0.19, *p* = 0.0369) and Akt phosphorylation (0.78 ± 0.061, *p* = 0.0074) were reduced in SMA cells compared to the control condition (data not shown). LY294002 addition significantly decreased the ratio of Akt phosphorylation versus total Akt in both Control (+LY: 0.69 ± 0.074, *p* = 0.0011) and SMA ( + LY: 0.727 ± 0.094, *p* = 0.0143) cells, compared to the Control and SMA LY294002 untreated (-LY) conditions, respectively (Fig. 1a). LY294002 did not reduce total Akt protein in Control (0.93 ± 0.034, *p* = 0.0919) nor in SMA (0.96 ± 0.054, *p* = 0.518) cells, compared to their respective -LY controls (Fig. 1b). Analysis of SMN level revealed a significant reduction of this protein in LY294002-treated Control (+LY 0.67 ± 0.079, *p* = 0.0024) and SMA ( + LY 0.57 ± 0.10, *p* = 0.0033) conditions compared to their respective non-treated (-LY) controls (Fig. 1b). To evaluate whether the SMN protein reduction observed was associated with decreased *SMN* gene expression, we quantified *SMN* messenger RNA (mRNA) by quantitative RT-PCR (qRT-PCR). Human *Gapdh* gene was used as a control. Seven-day differentiated human Control and SMA MNs were treated with or without 25 µm LY294002 during 24 h. To quantify *SMN* transcript level, total RNA was extracted and reverse-transcribed to cDNA. The results indicated a significant reduction in *SMN* mRNA expression in LY294002-treated conditions (+LY Control: 0.79 ± 0.065, *p* = 0.029; +LY SMA: 0.80 ± 0.048, *p* = 0.004) compared to non-treated (-LY) controls, suggesting that PI3K inhibition regulates SMN at transcriptional level in human Control and SMA MNs (Fig. 1c).

### ERK MAPK pathway regulates SMN in mouse and human MNs

**Fig. 2:**
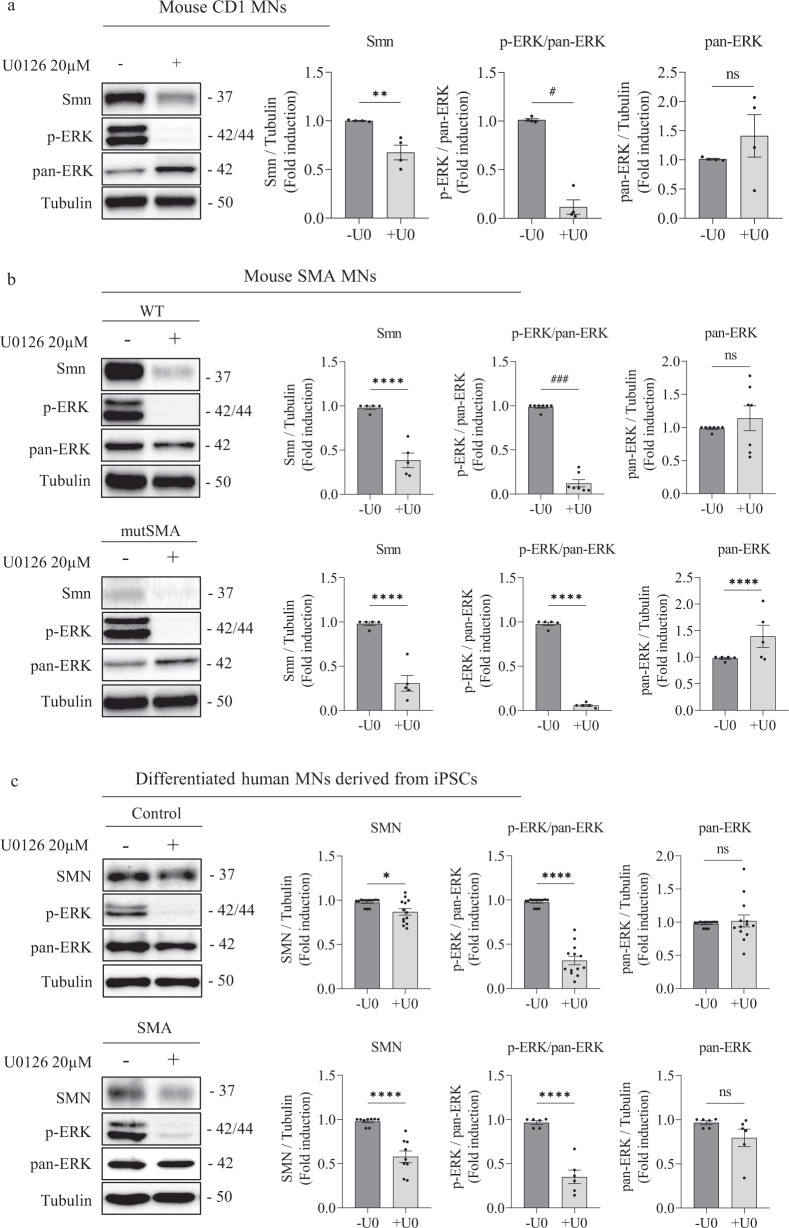
ERK MAPK pathway regulates SMN protein level in MNs. **a** Six-day CD1 cultured MNs, **b** 6-day WT (left) and mutSMA (right) cultured MNs, and **c** 7-day differentiated Control (left) and SMA (right) human MNs were washed and treated with 20 μM U0126 (U0) during 24 h or left untreated. Cell lysates were then obtained and submitted to western blot analysis using anti-phospho-ERK (p-ERK) or anti-SMN antibodies. Membranes were stripped and reprobed with anti-ERK (pan-ERK) antibody or with anti-α-tubulin antibody. Graphs represent the expression of Smn vs α-tubulin or p-ERK vs pan-ERK or pan-ERK vs α-tubulin, and correspond to the quantification of at least 4 independent experiments ± SEM. Symbols indicate differences using Student *t* test (**p < 0.01; ****p < 0.0001) or Mann-Whitney test (^#^*p* < 0.05; ^###^*p* < 0.001).

Previous results described an increased phosphorylation of ERK protein in SMA models, including MNs [14, 15]. To further evaluate the role of ERK MAPK pathway in SMN-reduced MNs, we studied the effect of ERK inhibition on SMN protein and mRNA using the MEK inhibitor U0126 (Cayman). Six-day cultured mouse (CD1; wild-type, WT; or Smn^−/−^; SMN2^+/+^, mutSMA) MNs and 7-day differentiated MNs from human iPSCs cells were treated with 20 µM U0126. At 24 h, cell lysates were obtained and submitted to western blot analysis using anti-phospho-ERK, anti-ERK, and anti-SMN antibodies (Fig. 2). When the U0126 inhibitor was added to the culture medium, the ratio of phospho-ERK (p-ERK) versus total ERK (pan-ERK) protein was significantly reduced in mouse (CD1 + U0, 0.116 ± 0.074, *p* = 0.0286; WT + U0, 0.122 ± 0.040, *p* = 0.0006; mutSMA +U0, 0.104 ± 0.318, *p* = 0.0005) (Fig. 2a, b) and human differentiated MNs (Control +U0, 0.316 ± 0.048, *p* < 0.0001, SMA + U0, 0.36 ± 0.100, *p* < 0.0001) (Fig. 2c), compared with the parallel untreated controls (-U0). No significant differences of total ERK protein level were observed in U0126 conditions compared to non-treated controls (CD1 *p* = 0.316; WT *p* = 0.429; mutSMA *p* = 0.0825; Control *p* = 0.652; SMA *p* = 0.1205) (Fig. 2). When analysing SMN protein levels, we observed a significant reduction in mouse and human U0126-treated conditions (mouse MNs: CD1 + U0, 0.675 ± 0.076, *p* = 0.0053; WT + U0, 0.385 ± 0.081, *p* < 0,0001, mutSMA +U0 0.306 ± 0.088, *p* < 0.0001; human differentiated MNs: Control +U0: 0.868 ± 0.037, *p* = 0.011, SMA + U0: 0.47 ± 0.054, *p* < 0.0001), compared to their respective untreated (-U0) controls (Fig. 2). These results indicated that ERK phosphorylation regulates SMN protein level in cultured MNs.

**Fig. 3:**
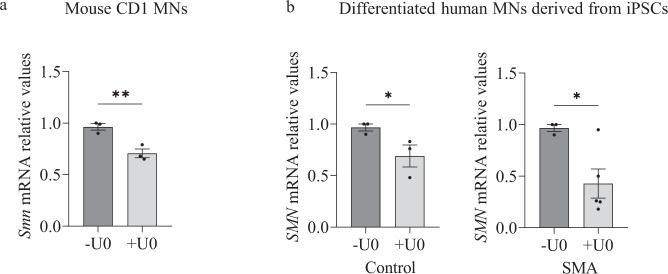
ERK MAPK pathway regulate *SMN* mRNA levels in MNs. Total RNA was extracted from 20 μM U0126-treated (+U0) or non-treated (-U0) (**a**) 6-day cultured CD1 MNs or (**b**) 7-day differentiated Control and SMA human MNs. RNA was reverse transcribed to cDNA. *Smn* (**a**) and *SMN* (**b**) were amplified through RT-qPCR. *Gapdh* (**a**) and *GAPDH* (**b**) were used as control. Graph values are the mean of *Smn* (**a**) and *SMN* (**b**) gene expression from 3 independent differentiation cycles ± SEM. Asterisks indicate significant differences using Student *t* test (*p < 0.05; **p < 0.01).

To validate that SMN protein decrease was related to reduced activity of *SMN* gene expression in U0126 treated cells, we quantified *Smn* mRNA by qRT-PCR, using *Gapdh* gene as a control in CD1 mouse MNs. Six-day cultured CD1 MNs were incubated with or without 20 µM U0126. At 20 h, total RNA was extracted for reverse transcription and amplification of *Smn* transcript. U0126 addition was associated with a significant reduction in *Smn* mRNA expression (0.73 ± 0.055, *p* = 0.042) in CD1 MNs, compared to the non-treated controls (Fig. 3a). Next, we measured *SMN* mRNA in Control and SMA cells in human differentiated MNs. Seven-day human MNs were treated with or without 20 µM U0126. After 24 h, total RNA was extracted and reverse-transcribed to cDNA, as a template to quantify *SMN* transcript level. *Gapdh* gene was used as a control. The results indicated a significant reduction in *SMN* mRNA expression in U0126-treated cultures (Control +U0, 0.69 ± 0.107, *p* = 0.045; SMA + U0, 0.43 ± 0.18, *p* = 0.023) compared to control non-treated conditions (Fig. 3b). Overall, these results show that ERK MAPK pathway inhibition regulated SMN at protein and transcriptional level in mouse and human cultured MNs.

### ERK inhibition regulates autophagy markers in human SMA MNs

**Fig. 4:**
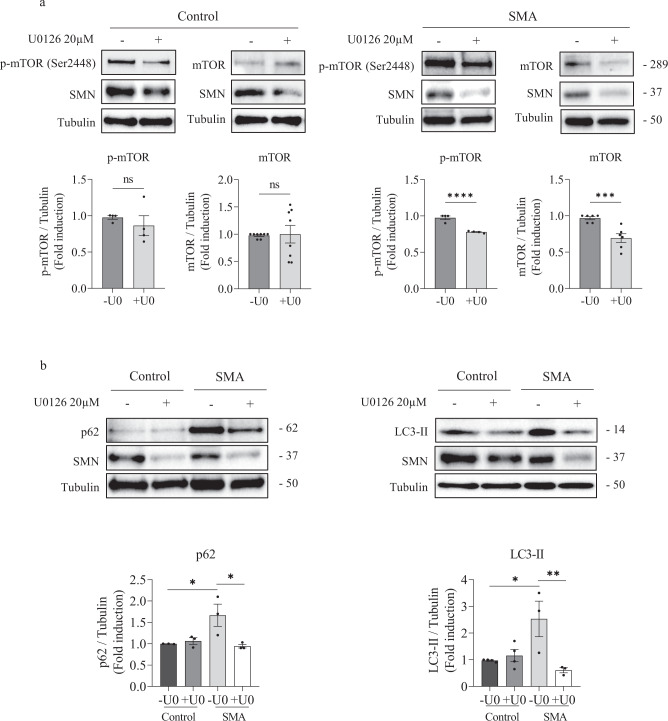
ERK MAPK inhibition regulates mTOR phosphorylation and autophagy markers in human SMA MN. **a**, **b** Seven-day differentiated Control and SMA human MNs were treated with 20 μM U0126 (U0) during 24 h or left untreated. Cell lysates were then obtained and submitted to western blot analysis using anti-phospho-mTOR (p-mTOR Ser2448), anti-mTOR, anti-p62, anti-SMN, or anti-LC3 antibodies. Membranes were reprobed with anti-α-tubulin antibody (**a**, **b**). Graphs represent the expression of p-mTOR or mTOR vs α-tubulin (**a**), and the expression of p62 or LC3-II vs α-tubulin (**b**), corresponding to the quantification of at least 3 independent iPSCs differentiation cycles ± SEM. Asterisks indicate differences using Student *t*-test (*p < *0.05;* ***p < 0.001; ****p < 0.0001; ns no significant differences *p* > 0.05).

Among other functions, ERK protein participates in the activation of mTOR protein, a well-known regulator of the autophagy process [19]. In this context, we wanted to analyze the effect of ERK phosphorylation inhibition on the level of autophagy markers in MNs. Seven-day differentiated human Control and SMA MNs were treated with 20 µM U0126. After 24 h, protein extracts were submitted to western blot analysis using anti-phospho(Ser2448)-mTOR (p-mTOR) and anti-mTOR (mTOR) antibodies. We observed no differences in p-mTOR (+U0 0.86 ± 0.137, *p* = 0.359) or mTOR (+U0 0.999 ± 0.16, *p* = 0.9983) protein level in U0126-treated Control cultures, compared to their respective untreated controls (Fig. 4a). In contrast, U0126 addition to SMA cells induced a significant reduction in p-mTOR (+U0 0.77 ± 0.007, *p* < 0.0001) and mTOR (+U0 0.69 ± 0.06, *p* < 0.0001) protein levels, compared with the non-treated condition (-U0) (Fig. 4a). Inhibition of ERK phosphorylation was confirmed by blotting p-ERK and pan-ERK antibodies.

Next, we analyzed the levels of LC3 and p62 autophagy markers. Control and SMA MNs were differentiated and treated with U0126 as described above. Protein extracts were submitted to western blot analysis using anti- p62 or anti- LC3 antibodies. The level of p62 and LC3-II proteins increased in SMA (p62 1.667 ± 0.26, *p* = 0.035; LC3-II 2.53 ± 0.66, *p* = 0.0099) MNs, compared to Control cells (Fig. 4b). In SMA condition, U0126 treatment significantly reduced p62 and LC3-II protein levels, compared to their untreated controls (p62: SMA -U0: 1.667 ± 0.26; SMA + U0: 0.943 ± 0.04, *p* = 0.024; LC3-II: SMA -U0: 2.53 ± 0.66; SMA + U0: 0.606 ± 0.094, *p* = 0.0259). However, Control cultures showed no differences in p62 and LC3-II protein levels in U0126-treated (+U0) cells compared with their respective non-treated controls (-U0) (p62, *p* = 0.98; LC3-II, *p* = 0.5236) (Fig. 4b). Together these results indicated that ERK inhibition reduces mTOR protein and autophagy markers in human SMN-reduced MNs, but not in Control cells.

### SMN overexpression does not regulate ERK phosphorylation in human differentiated SMA MNs

**Fig. 5:**
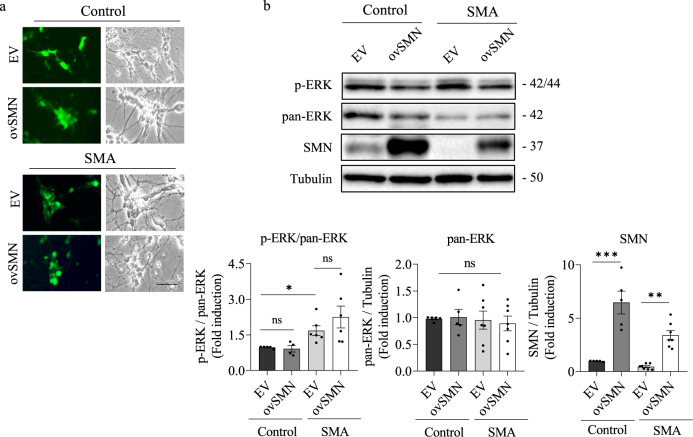
SMN overexpression does not prevent ERK hyperphosphorylation in SMA MNs. Control and SMA human MNs were plated and transduced with lentivirus containing the empty vector (EV) or the ovSMN construct and cultured for 7 days. **a** Representative microscopy images of 7-day transduced cultures. GFP positivity (left panel) indicates lentivirus transduced cells in the culture dish. Scale bar, 60 μm. **b** Protein extracts of transduced MN cultures were submitted to western blot analysis and probed with anti-p-ERK or anti-SMN antibodies. Membranes were stripped and reprobed with anti-pan-ERK antibody or with anti-α-tubulin antibody. Graphs represent the expression of p-ERK vs pan-ERK, pan-ERK vs α-tubulin, and SMN vs α-tubulin, and correspond to the quantification of 4 independent iPSCs differentiation cycles ± SEM. Asterisks indicate significant differences using Student *t* test (*p < 0.05) or one-way ANOVA with Tukey’s multiple comparisons test (**p < 0.005; ****p < 0.0001; ns no significant differences *p* > 0.05).

To evaluate whether the increase in SMN protein levels regulates ERK protein and phosphorylation in SMA MNs, we used lentiviral RNA vectors to overexpress SMN in these cells. To this end, Control and SMA human MNs were plated and transduced with lentivirus containing the empty vector (EV) control or SMN overexpression (ovSMN) constructs. Twenty-four hours later, lentivirus medium was replaced with fresh culture medium and cells were maintained in these conditions for 7 days. Lentivirus transduction efficiency was microscopically monitored by observing green fluorescent protein (GFP) positive cells (Fig. 5). Cell lysates were obtained and subjected to immunoblot analysis using anti-p-ERK, anti-pan-ERK, and anti-SMN antibodies. SMN protein level was significantly increased in ovSMN Control (6.46 ± 1.067, *p* < 0.0001) and SMA (3.40 ± 0.439, *p* = 0.0014) cultures, compared to the respective EV controls (Fig. 5). In agreement with previous results [14], p-ERK/pan-ERK ratio was increased in SMA (1.681 ± 0.205, *p* = 0.0292) cultures compared to the Control condition. Nevertheless, SMN overexpression did not induce significant differences in the p-ERK/pan-ERK ratio in Control (*p* = 0.5871) or SMA (*p* = 0.2822) ovSMN cultures, compared to EV controls (Fig. 5). These results indicate that in SMA MNs, SMN increase did not reduce ERK phosphorylation to the basal level observed in the control cells.

### Intracellular calcium chelation prevents increased ERK phosphorylation in human SMA MNs

**Fig. 6:**
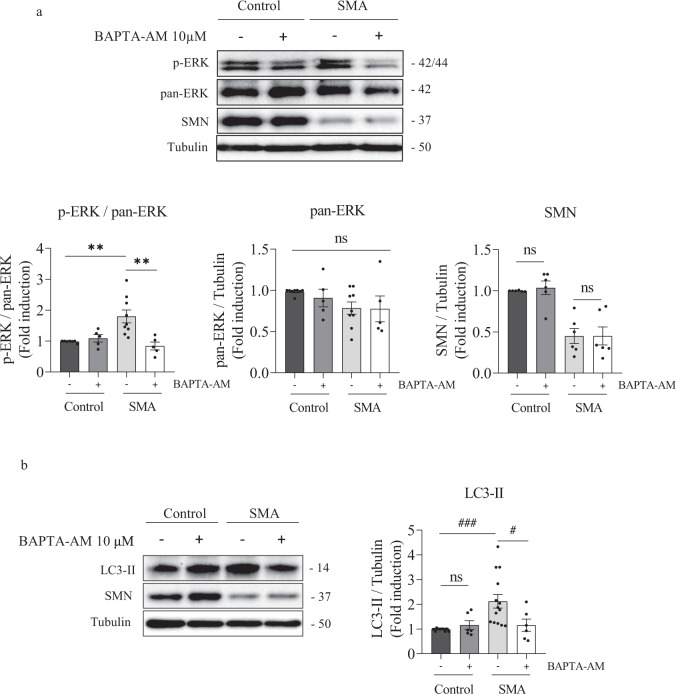
Calcium chelator BAPTA-AM prevents ERK phosphorylation and reduces LC3-II level in SMA human differentiated MNs. Seven-day differentiated Control and SMA human MN cultures were washed and treated with 10 μM BAPTA-AM or left untreated. At 24 h, protein extracts were obtained and submitted to western blot analysis using anti-p-ERK (**a**), anti-LC3 (**b**), or anti-SMN (**a**, **b**) antibodies. Membranes were stripped and reprobed with anti-pan-ERK antibody or with anti-α-tubulin antibody. Graphs represent the expression of p-ERK vs pan-ERK (**a**), or ERK (**a**) or SMN (**a**) or LC3-II (**b**) vs α-tubulin, corresponding to the quantification of at least 3 independent iPSCs differentiation cycles ± SEM. Symbols indicate differences using one-way ANOVA with Tukey’s multiple comparisons post-test (**p < 0.01) or Kruskal-Wallis with Dunn’s multiple comparisons post-test (^#^*p* < 0.05; ^###^*p* < 0.001); (ns no significant differences *p* > 0.05).

To elucidate the intracellular mechanisms involved in ERK phosphorylation increase observed in SMA MNs, we examined the effect of reducing intracellular calcium level using the calcium chelator BAPTA. To this end, 7-day Control and SMA human differentiated MNs were treated or not with 10 µM BAPTA (BAPTA-AM, Invitrogen). At 24 h, protein extracts were obtained and submitted to western blot using anti-SMN, anti-p-ERK, and anti-pan-ERK antibodies. In Fig. 6, results indicate that ERK phosphorylation was significantly increased in SMA (p-ERK/pan-ERK ratio 1.796 ± 0.20, *p* = 0.0037) cells compared with the Control MNs (Fig. 6). BAPTA addition significantly reduced p-ERK/pan-ERK ratio in SMA (0.84 ± 0.12, *p* = 0.002) MNs but not in Control (1.087 ± 0.119, *p* = 0.97) MNs, compared with their untreated respective controls (Fig. 6a). BAPTA treatment did not modify SMN protein level in Control (*p* = 0.987) and SMA (*p* = 0.999) MNs compared to their respective untreated controls.

Finally, we analyzed the effect of intracellular calcium reduction on the autophagy marker LC3-II. To this aim, 7-day differentiated Control and SMA human MNs were treated with 10 µM of BAPTA-AM during 24 h. Protein extracts were obtained and submitted to western blot analysis with anti-LC3, anti-p-ERK and anti-SMN antibodies (Fig. 6b). As expected, LC3-II levels were increased in SMA (2.122 ± 0.27, *p* = 0.0004) MNs, compared to the Control condition. BAPTA-AM treatment significantly reduced LC3-II protein level in SMA ( + BAPTA-AM 1.165 ± 0.25, *p* = 0.042) cultures compared to SMA non-treated (-BAPTA-AM 2.122 ± 0.27) cells. In Control cultures, no differences on LC3-II protein level were observed between BAPTA-AM-treated and untreated cells (*p* > 0.99). These results together indicate that intracellular calcium reduction prevented ERK hyperphosphorylation and decreased LC3-II autophagy marker in SMA human differentiated MNs.

## Discussion

The precise intracellular mechanisms causing MN degeneration in SMA disease are not fully understood. Here, we report a link between ERK signaling pathway alteration and autophagy deregulation in SMA MNs. The activity of ERK MAPK and PI3K/Akt pathways is essential for the survival and maintenance of several neuronal types, including MNs [9]. SMA cells exhibit reduced Akt phosphorylation [14, 16, 23]. On the other hand, increased ERK phosphorylation has been described in mouse SMA spinal cord and tibialis anterior muscle [16, 23], in SMA astrocytes derived from human iPSCs [24], in SMN-reduced motoneuron-like NSC34 cells [15], and in cultured mouse and human SMA MNs [14]. To explain ERK hyperactivation in SMN-reduced cells, a compensatory positive effect of this hyperactivity has been proposed as preventing MN degeneration, depending on the cellular context [15]. Additionally, our results indicate that PI3K and ERK inhibition regulated *SMN* at transcriptional level. Both PI3K/Akt and ERK MAPK pathways have been linked to the positive regulation of *SMN* transcription through Akt/CREB and ERK/Elk-1, respectively [23, 25]. Accordingly, our results show that the inhibition of either PI3K or MEK produces a reduction in SMN protein and mRNA levels in non-SMA and SMA cultured MNs. Nonetheless, the effects of ERK inhibition within SMA models are controversial. Previous studies demonstrated a beneficial effect of ERK inhibition on SMN expression in the spinal cord of SMA-like mice and in human SMA myotubes, along with lifespan extension in a severe SMA mouse model [16]. Conversely, the inhibition of ERK phosphorylation aggravates the SMA phenotype in the Taiwanese SMA mouse model and induces cell death, specifically in MNs, despite a protective effect in total primary spinal cord cultures and NSC34 cells [15]. Our observations showed SMN reduction after ERK inhibition in cultured mouse and human MNs, suggesting that ERK activity could regulate SMN transcription in these cells. SMA is considered a multi-systemic pathology in which multiple cell types can be diversely affected depending on the disease genotype [26]. For instance, a transcriptional study of muscle tissue from patients with severe and mild SMA revealed differences in the expression of proteins involved in cell survival and autophagy between the two disease types [27]. Hence, further analysis of ERK phosphorylation and SMN regulation in SMA tissues and genotypes could provide new insights about disease evolution.

Previous findings in SMA cells suggest increased autophagy markers and mTOR phosphorylation, correlating with the increase of autophagosome formation and reduced autophagic flux [28]. Our current results indicate that pharmacological inhibition of ERK pathway reduced mTOR phosphorylation and p62 and LC3-II protein levels in SMA differentiated MNs, but not in control cells. ERK is a positive regulator of mTORC1 signaling [19, 20], which in turn participates in autophagy inhibition [29, 30]. Altogether, these outcomes suggest that the alterations observed in SMA MNs regarding mTOR and autophagy markers may be related to a further alteration of the ERK MAPK signaling pathway.

The exact mechanism(s) by which ERK phosphorylation is increased in SMA cells remains unclear. Although ERK appears to be linked to SMN transcriptional regulation, our results suggested that SMN increase does not regulate ERK phosphorylation in human Control and SMA MNs (Fig. 5) or in CD1 MNs (unpublished results). These observations appear to rule out a direct link between SMN levels and ERK activity and to suggest that ERK signaling pathway hyperphosphorylation may be a secondary consequence of SMN reduction. Interestingly, intracellular calcium levels have been reported to be upstream regulators of ERK pathway, promoting its activation [31]. Using the intracellular calcium chelator BAPTA-AM, we showed that the ERK hyperphosphorylation and increased LC3-II level are prevented in SMA MNs, suggesting that intracellular calcium may regulate ERK signaling and autophagy in these cells. Intracellular free calcium could be increased in neurons of patients who develop MN diseases such as amyotrophic lateral sclerosis [32]. In SMN-reduced MNs, calcium homeostasis is altered in nerve terminals and presynaptic mitochondria [33, 34], suggesting that changes in intracellular calcium may modulate neurotransmission and intracellular pathways in SMA cells. Additionally, disrupted calcium signaling has been observed in SMA iPSCs-derived astrocytes. These cells display increased basal calcium level and enhanced ERK activation compared to the control [24].

**Fig. 7:**
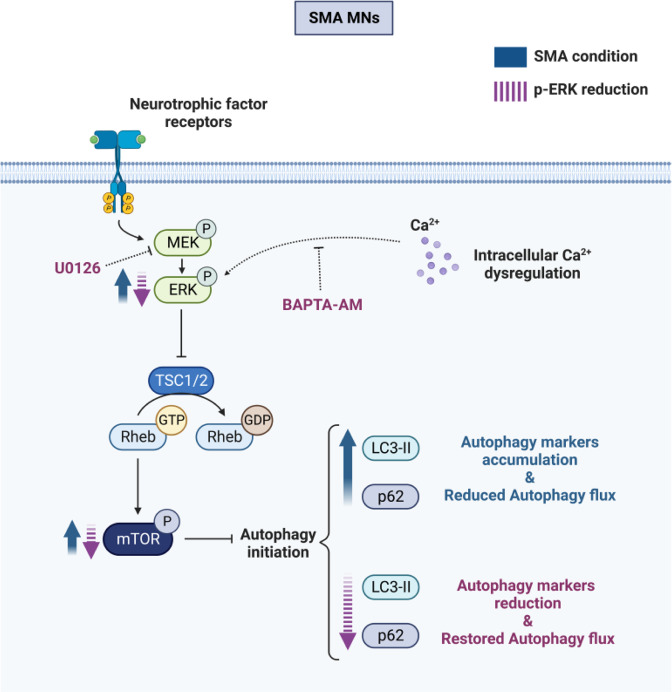
Proposed mechanisms involved in ERK and autophagy pathways regulation in SMN-reduced MNs. In SMA condition, ERK and mTOR phosphorylation had increased in MNs, together with an increase of the autophagy markers LC3-II and p62, suggesting a reduction of the autophagic flux. ERK MAPK pharmacological inhibition or reduction of intracellular calcium levels prevented ERK phosphorylation, reduced mTOR phosphorylation, and restored autophagy markers to a level comparable to non-SMA MNs (created with BioRender.com).

Overall, these results suggest a connection between intracellular calcium levels and the activity of ERK signaling pathway, which in turn may regulate autophagy and SMN in SMA MNs (Fig. 7). Nonetheless, whether ERK overactivation is beneficial or deleterious in SMA disease is yet to be determined and can have diverse consequences depending on SMN requirements [15]. Modulation of ERK pathway may constitute a new strategy to target MN degeneration by regulating SMN protein and the autophagy process in SMA cells.

## Materials and methods

### SMA animals

The severe SMA mouse model FVB·Cg-Tg (SMN2)^89Ahmb^Smn1^tm1Msd^/J (The Jackson Laboratory, Bar Harbor, Maine, USA) was used for experimental procedures. Heterozygous females and males were crossed to obtain Smn^−/−^; SMN2^+/+^ (hereafter referred to as mutSMA) and Smn^+/+^; SMN2^+/+^ (wild-type, hereafter referred to as WT) mice.

A piece of the tail from neonatal offspring was collected for genotyping. The REDExtract-N-Amp Tissue PCR Kit (Sigma, Saint Louis, MO, USA) was used for genomic DNA extraction and PCR setup, with the following primers: WT forward 5′-CTCCGGGATATTGGGATTG-3′, SMA reverse 5′-GGTAACGCCAGGGTTTTCC-3′ and WT reverse 5′-TTTCTTCTGGCTGTGCCTTT-3′.

All procedures were done in accordance with the Spanish Council on Animal Care guidelines and approved by the University of Lleida Advisory Committee on Animal Services (CEEA02–01/17).

### Spinal cord MN isolation and culture

CD1 or SMA embryonic 13 (E13) mice were used for spinal cord MN primary cultures. Cells were isolated and plated in laminin-coated four-well dishes (Nunc, Thermo Fisher Scientific, Waltham, MA, USA) as described [35, 36].

Culture medium was NBM complete (NBMc): neurobasal medium (Gibco, Thermo Fisher Scientific) supplemented with B27 (2% *v*/v; Gibco), horse serum (2% v/v; Gibco), l-glutamine (0.5 mM; Gibco) and 2-mercaptoethanol (25 μM; Sigma), and a cocktail of neurotrophic factors (1 ng/ml BDNF, 10 ng/ml GDNF, 10 ng/ml CT-1, and 10 ng/ml HGF, all from Peprotech, London, UK). At 24 h after plating, 2 μg/ml of aphidicolin (Sigma) was added to the culture medium and was maintained throughout the experiment.

### Differentiation of human-induced pluripotent stem cells (iPSCs) to MNs

The human iPSCs were acquired from Coriell Institute for Medical Research (Camden, NJ, USA). The GM23411*B iPSC cell line (healthy non-fetal tissue, 3 months old individual at sampling) was used as a control (Control) and GM23240*B iPSC cell line (SMA) was from a patient with SMA type II (*SMN2* 2 copies; delta exon7–8 in *SMN1*; 3 years old individual at sampling). SMA patient was previously classified as SMA type I, but several data supported re-classification to SMA type II. Control and SMA cells were differentiated to MNs as described [37, 38]. Briefly, iPSCs were cultured and expanded on Geltrex coated plates (Gibco) in Essential 8 medium (Gibco). Cells were dissociated with Accutase (Gibco) and plated in neuroepithelial induction medium (NEPIM: DM/F12:NBMPlus 1:1 supplemented with B27, l-glutamine, and NEAA [all from Gibco]; 0.1 mM ascorbic acid [Sigma]; and 3 μM CHIR99021; 2 μM SB431512; and 2 μM DMH1 [all from Cayman, Ann Arbor, MI, USA]) to generate neuroepithelial (NEP) cells. After six days in vitro, NEP cells were dissociated and expanded with NEPIM containing 0.1 μM retinoic acid (Sigma), 0.5 μM purmorphamine (Cayman) and 0.5 mM valproic acid (Sigma) to produce MN progenitors (MNPs). Next, MNPs were detached and cultured in MN induction medium (NEPIM plus 0.5 μM retinoic acid, 0.1 μM purmorphamine) to generate neurospheres. After six days, neurospheres were dissociated and plated on laminin-coated dishes in MN maturation medium (MN induction medium supplemented with 0.1 μM Compound E [Sigma], and 20 ng/ml CNTF, and 20 ng/ml IGF-1, both from Peprotech) to induce MN differentiation.

For experiments, dissociated neurospheres were plated in the following conditions: 80,000 cells/well in laminin-coated four-well tissue culture dishes (Nunc, Thermo Fisher Scientific) for western blot analysis, 500,000 cells/well in laminin-coated six-well tissue-culture dishes (Falcon, Corning Incorporated, Corning, NY, USA) for qRT-PCR experiments, or 15,000 cells/well on 1 cm^2^ laminin-coated glass coverslips placed into four-well dishes for immunofluorescence experiments. Independent Control and SMA iPSCs differentiation cycles were used for replicates of the experiments. Confocal images were obtained using FV10i Olympus microscope (Tokyo, Japan) and for cell area analysis (blinded) the NIH ImageJ software was used.

### Plasmids and production of lentiviral particles

For SMN overexpression, the open reading frame of the human *SMN1* cDNA (NCBI accession number NM000344) was subcloned into FCIV plasmid and lentivirus containing overexpression constructs was obtained as described [35]. For lentiviral transduction, cells were plated in four-well dishes; the medium containing lentivirus was added 3 h later. The medium was changed 20 h later and infection efficiency was monitored in each experiment by direct counting of GFP-positive cells. Overexpression efficiency was monitored by western blot analysis using an anti-SMN antibody (BD Transduction Laboratories, Franklin Lakes, NJ, USA).

### Western blot analysis

Western blots were performed as previously described [36]. Total cell lysates of cultured cells (60,000 cell/well) were resolved in sodium dodecyl-sulfate polyacrylamide gels and transferred onto polyvinylidene difluoride Immobilon-P transfer membrane filters (Millipore, Billerica, MA, USA) using an Amersham Biosciences semidry Trans-Blot (Buckinghamshire, UK). The membranes were blotted with the following: anti-SMN (1:5000, Cat. No. 610646, BD Biosciences), anti-phospho-Akt (Thr 308) (p-Akt, 1:1000, Cat. No. 9275, Cell Signaling Technology, Danvers, MA, USA), anti-Akt-1 (pan-Akt, 1:10000, Cat. No. SC-1618, Santa Cruz Biotechnology, Dallas, TX, USA), anti-phospho-p44/42 ERK1/2 Thr202/Tyr204 (p-ERK, 1:15000, Cat. No. 9101, Cell Signaling Technology), anti-ERK (pan-ERK, 1:5000, Cat. No. 612641, BD Biosciences), anti-LC3 (1:1000, Cat. No. 2775), anti-phospho-mTOR (Ser2448) (p-mTOR, 1:1000, Cat. No. 5536), anti-mTOR (1:1000, Cat. No. 2972), and anti-p62/SQSTM1 (1:1000, Cat. No. 5114), all from Cell Signaling Technology. Specific protein content per lane was assessed reprobing the membranes with monoclonal anti-α-tubulin antibody (1:50000, Cat. No. T5168, Sigma). Blots were developed using Immobilon® Western Chemiluminescent HRP Substrate (Millipore, Burlington, MA, USA).

### Immunofluorescence

Cultured cells were fixed with 4% paraformaldehyde (Sigma) for 10 min and with cold methanol (Sigma) for 10 additional min. Cells were permeabilized with 0.3% Triton X-100 and incubated for 30 min with 10% BSA in PBS. The primary antibody (anti-HB9 antibody, 1:75, Cat. No. ab92606 or anti-ChAT antibody, 1:100, Cat. No. ab18736, both from Abcam (Cambridge, UK); anti-Islet1/2 antibody, 1:50, Cat. No. 39.4D5, Developmental Studies Hybridoma Bank (Iowa City, IA, USA); or anti-beta-III-tubulin, 1:400, Cat. No. 5568, Cell Signaling Technology) was diluted in 0.3% Triton-X-100 and incubated overnight with 10% BSA in PBS. After washing, the secondary antibody was added: anti-mouse ALEXA555 antibody, 1:400, Cat. No. A21422 or anti-rabbit ALEXA488 antibody, 1:400, Cat. No. A11008 (both from Invitrogen, Waltham, MA, USA); or anti-sheep ALEXA649 antibody, 1:400, Cat. No. 713-496-147 (Jackson ImmunoResearch, West Grove, PA, USA). To identify nuclear localization in cells Hoechst (1:400, Sigma) staining was used. Samples were mounted using Mowiol (Calbiochem, San Diego, CA, USA) medium. Microscopy observations were performed in an Olympus FV10i confocal microscope.

### RNA isolation and quantitative RT-PCR

CD1 or human differentiated MNs were plated (400,000 cells/well) in laminin-coated six-well tissue-culture dishes (Falcon, Corning Incorporated). Once the cells were attached, MNs were treated with or without 25 μM LY294002 (LY) (Calbiochem, Sigma) or 20 μM U0126 (U0) (Cayman Chemical) for 24 h. Total RNA was extracted using the RNeasy® Mini Kit (Qiagen, Hilden, Germany). Eighty nanograms of total RNA from each condition were used for each individual qRT-PCR reaction. The assays were performed in a CFX96 Real-Time System (Bio-Rad, Hercules, CA, USA) using iTaq™ Universal SYBR® Green One-Step Kit from Bio-Rad. For CD1 experiments, Real-Time was performed using mouse *Smn*-specific primers: *Smn* exon 1-forward (5′-GATGATTCTGACATTTGGGATG-3′) and *Smn* Exon 2-reverse (5′-TGGCTTATCTGGAGTTTCAGAA-3′) and specific primers of mouse glyceraldehyde-3-phosphate dehydrogenase (*Gapdh*): Forward (5′-TGCACCACCAACTGCTTAG-3′) and reverse (5′-GGATGCAGGGATGATGTTC-3′) as internal control. For human differentiated MNs, Real-Time was performed using human *SMN*-specific primers: *SMN* exon 6-forward (5′-CCGCCACCCCCTCCCATCTCT-3′) and *SMN* Exon 8-reverse (5′- CATCTCCTGAGACAGAGCTGA-3′) and specific primers of human glyceraldehyde-3-phosphate dehydrogenase (*GAPDH*): forward (5′- TGCACCACCAACTGCTTAG-3′) and reverse (5′-AGAGGCAGGGATGATGTTG-3′) as internal control. Quantification was completed using Bio-Rad CFX Manager real-time detection system software (version 3.1, Bio-Rad). Each sample was measured in triplicate; relative fold change gene expression levels were calculated with the formula 2^−(ΔΔCq).

### Statistical analysis

All experiments were performed at least three independent times (from three separate mouse offspring for primary MN cultures or three separate iPSCs differentiation cycles). Values were expressed as mean ± estimated standard error of the mean (SEM). Statistical analysis was performed using GraphPad Prism, version 9.4.1 (GraphPad Software Inc, San Diego, CA, USA). Normality of the data was tested with Shapiro-Wilk test. If the data distribution was normal, differences between two groups were assessed by two-tailed Student *t-*test, and differences between more than two groups one-way ANOVA with Tukey’s multiple comparisons test was used. If the data was not following a normal distribution, the non-parametric two-tailed Mann-Whitney test was applied to compare differences between two groups, and for more than two groups the non-parametric Kruskal-Wallis with Dunn’s multiple comparisons test was performed. Values were considered significant when *p* < 0.05. Statistical significance is shown with “*” when a parametric test was applied and with “#” when a non-parametric test was applied.

## Supplementary information

Original western blot

## Data Availability

The experimental data sets generated and/or analyzed during the current study are available from the corresponding author upon reasonable request. No applicable resources were generated during the current study.
