# Supplementary material for: ERK MAPK signaling pathway inhibition as a potential target to prevent autophagy alterations in Spinal Muscular Atrophy motoneurons
Source: Cell Death Discov. 2023 Apr 5;9:113. doi: 10.1038/s41420-023-01409-x (PMC10076363; doi:10.1038/s41420-023-01409-x)

Full and uncropped western blot for Fig 1b, left panel - Control

Lanes 1, 2 are on the figure

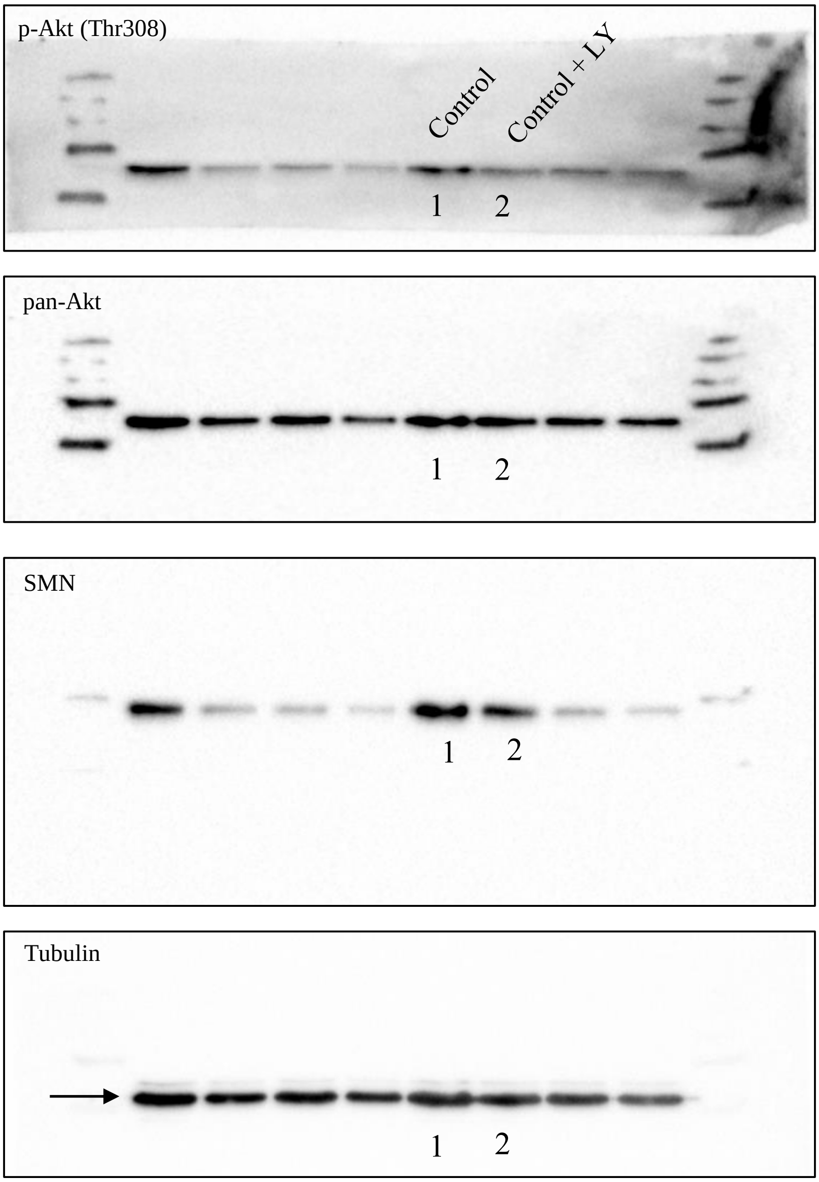

Full and uncropped western blot for Fig 1b, right panel - SMA

Lanes 1, 2 are on the figure

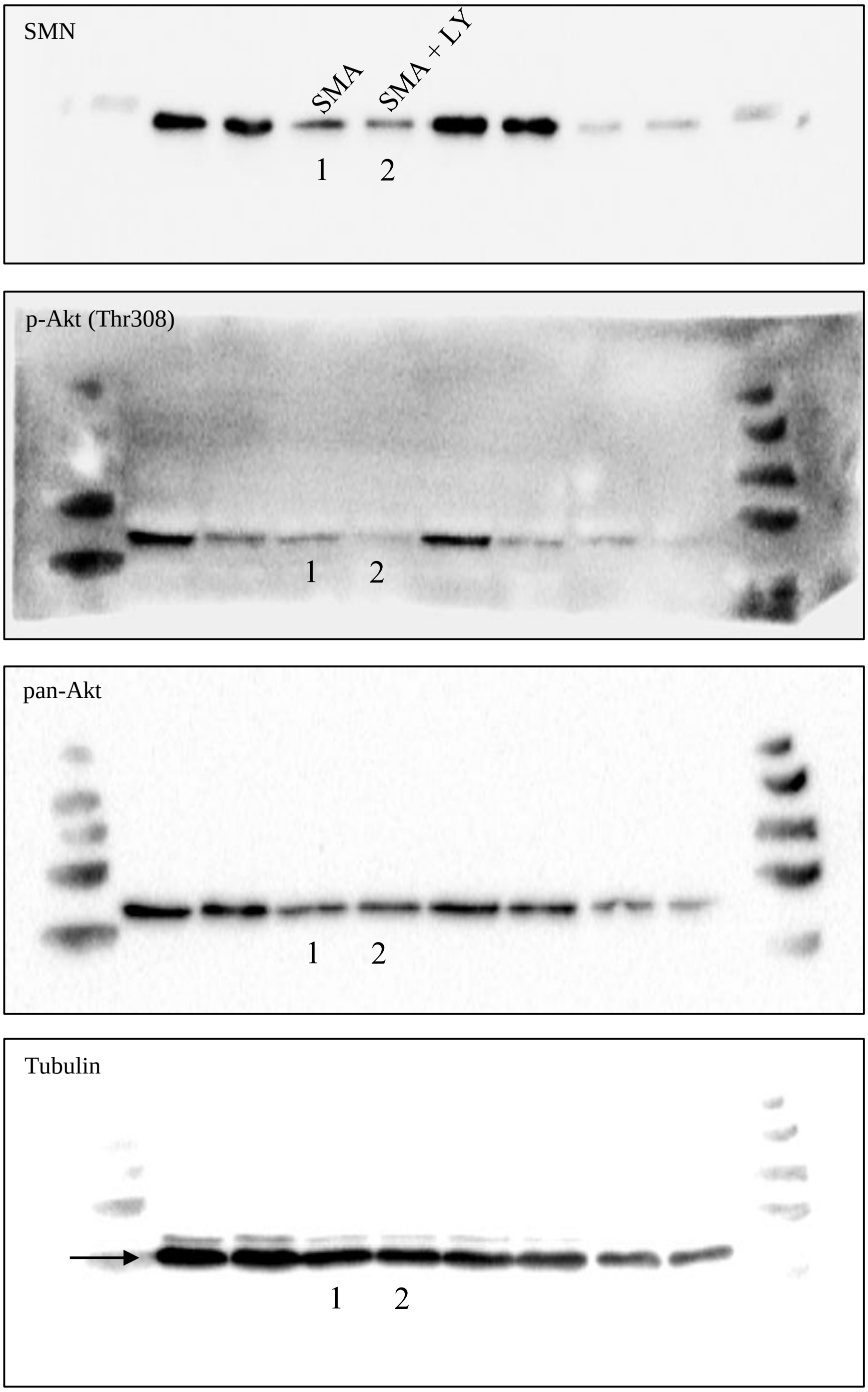

Full and uncropped western blot for Fig 2a

Lanes 1, 2 are on the figure

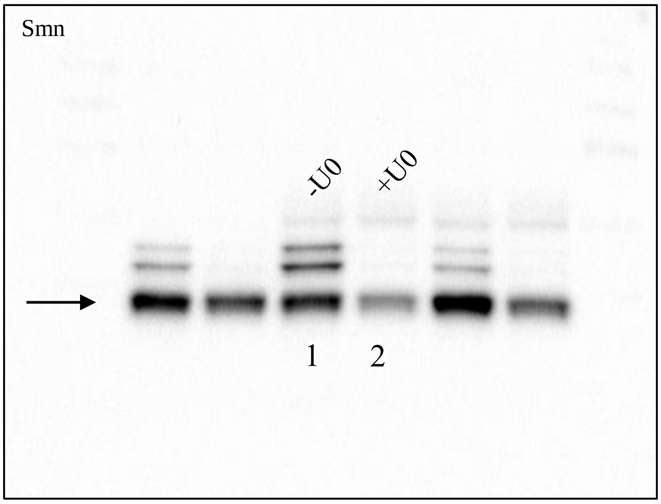

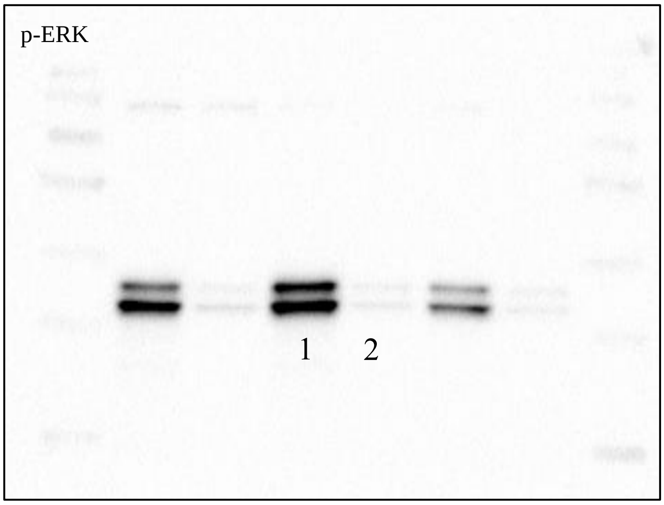

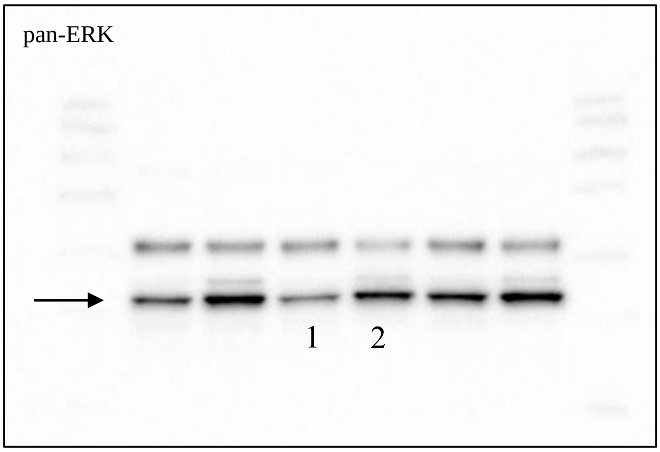

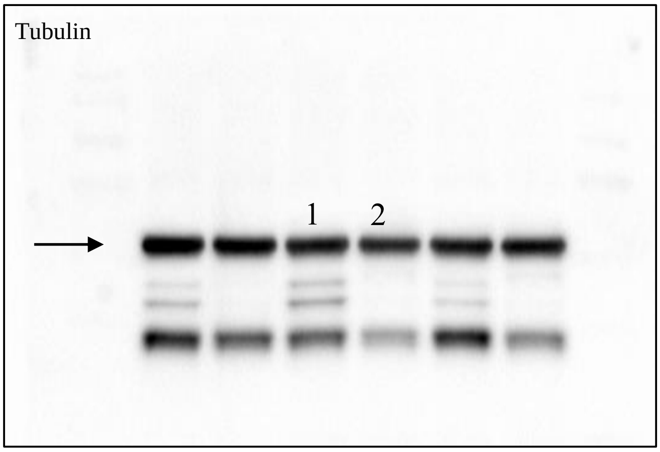

Full and uncropped western blot for Fig 2b

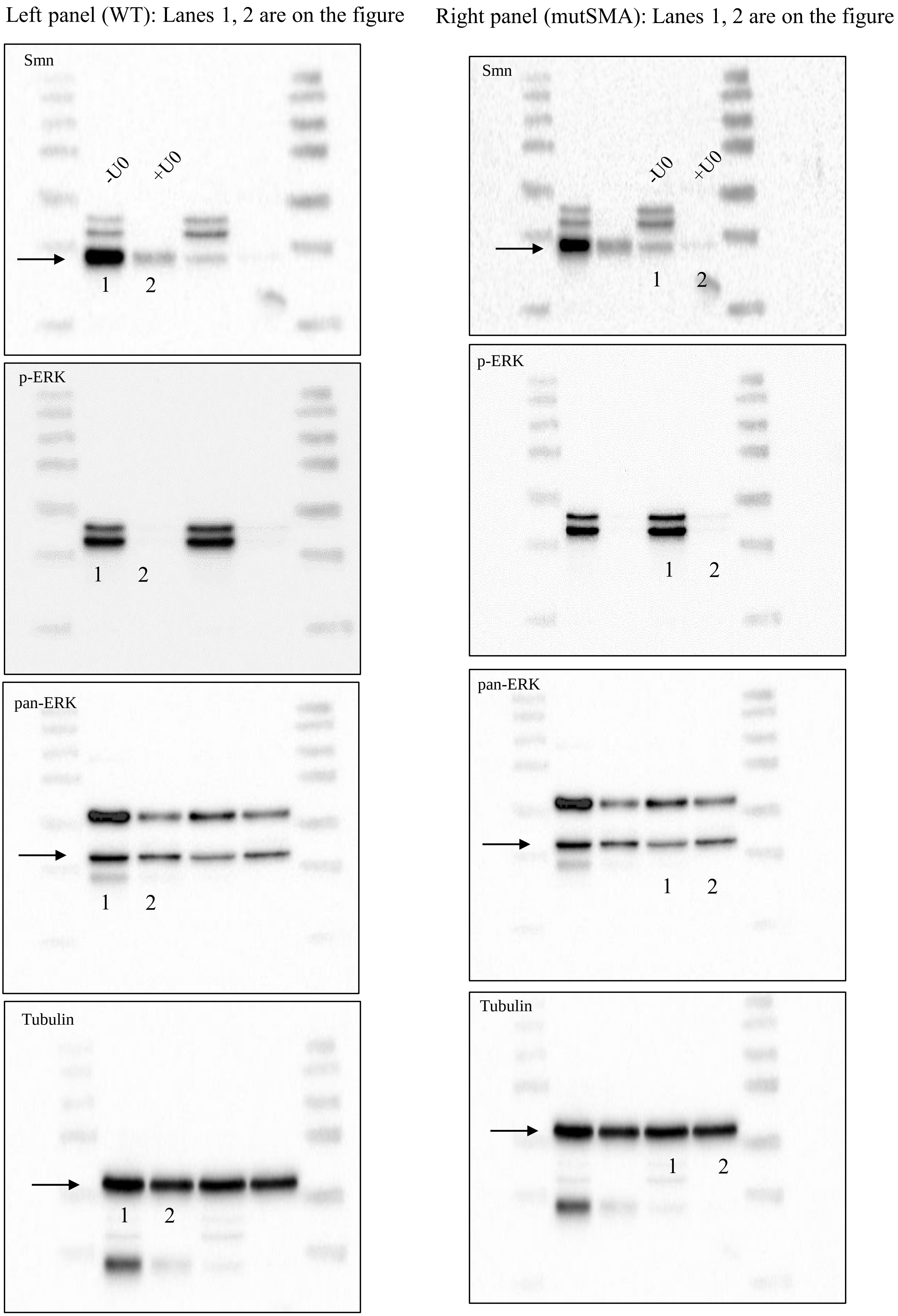

Full and uncropped western blot for Fig 2c

Left panel (Control): Lanes 1, 2 are on the figure

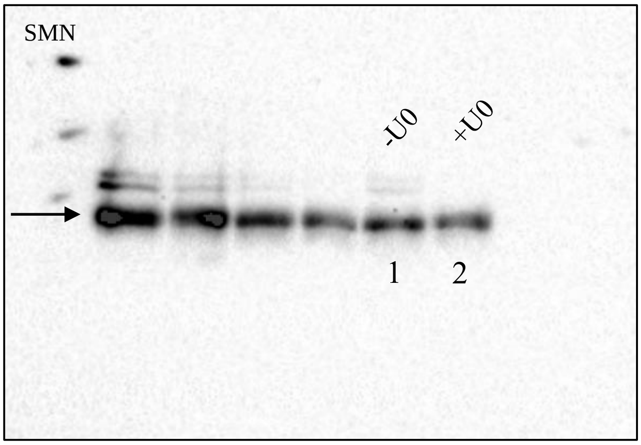

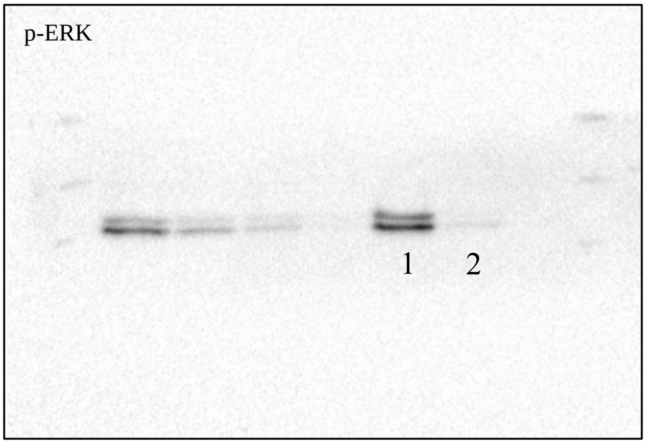

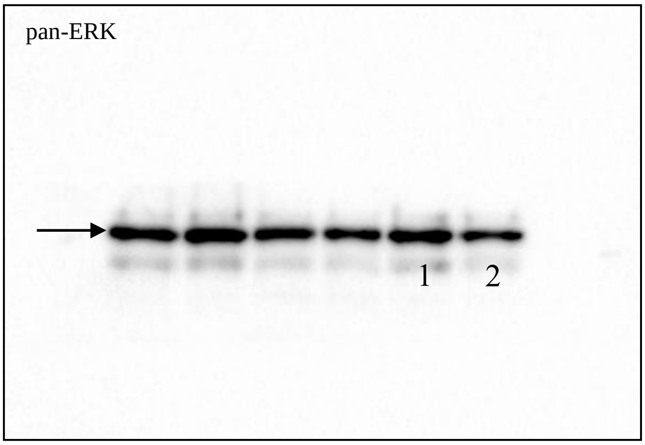

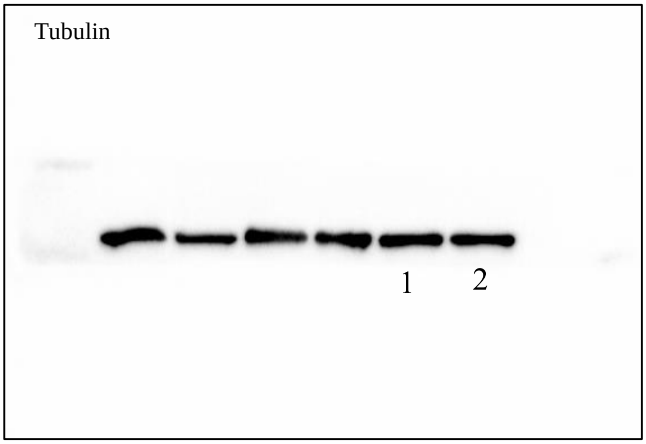

Left panel (SMA): Lanes 1, 2 are on the figure

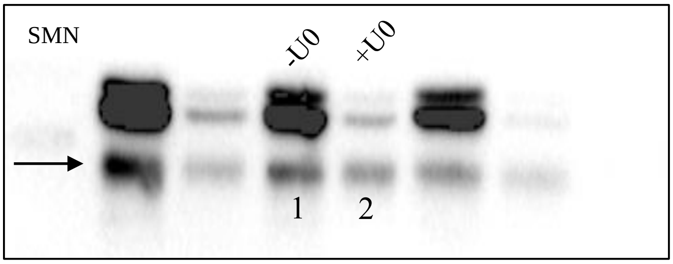

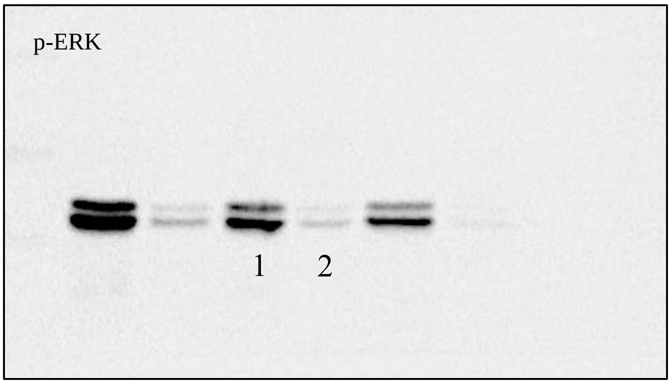

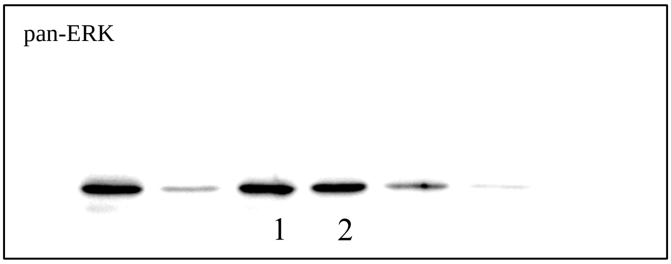

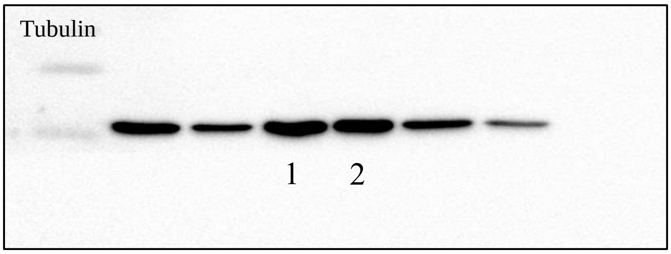

Full and uncropped western blot for Fig 4a Left panel - Control

p-mTOR (Ser2448): Lanes 1, 2 are on the figure

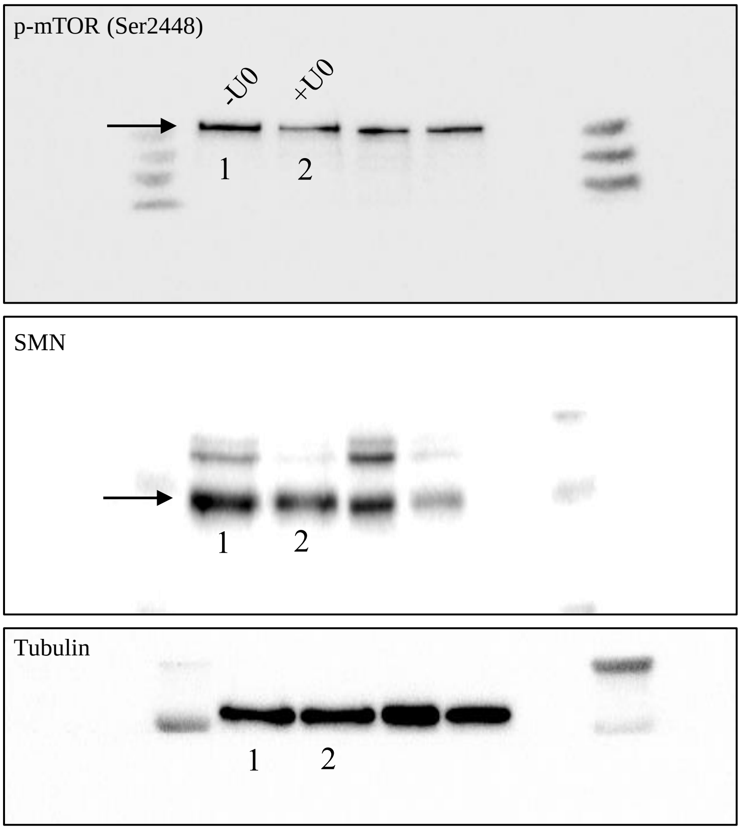

mTOR: Lanes 1, 2 are on the figure

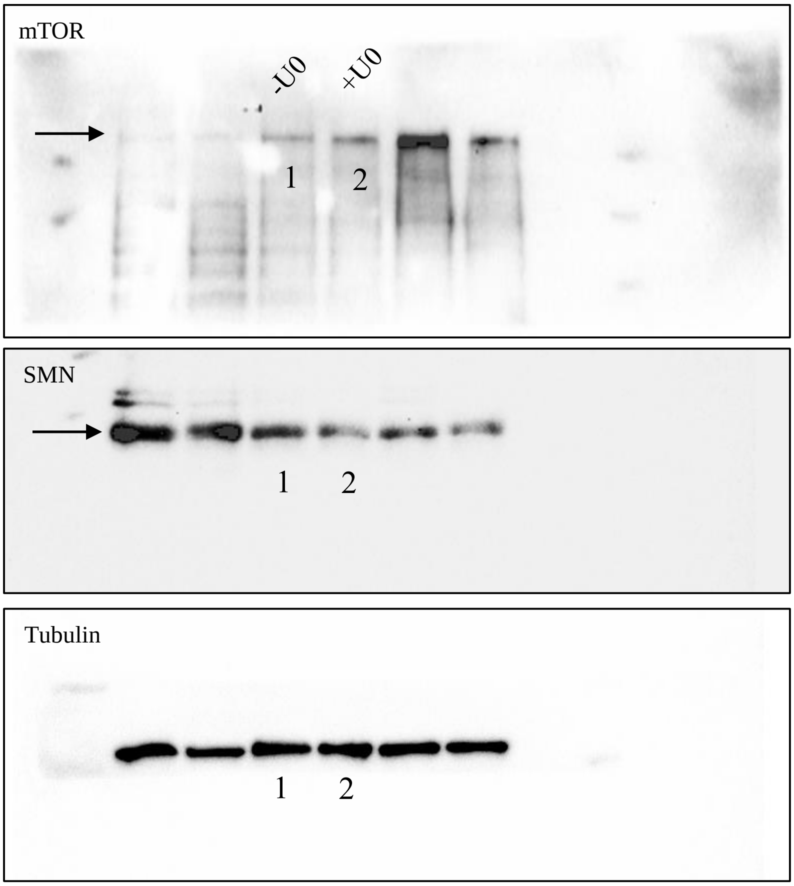

Full and uncropped western blot for Fig 4a Right panel - SMA

p-mTOR (Ser2448): Lanes 1, 2 are on the figure

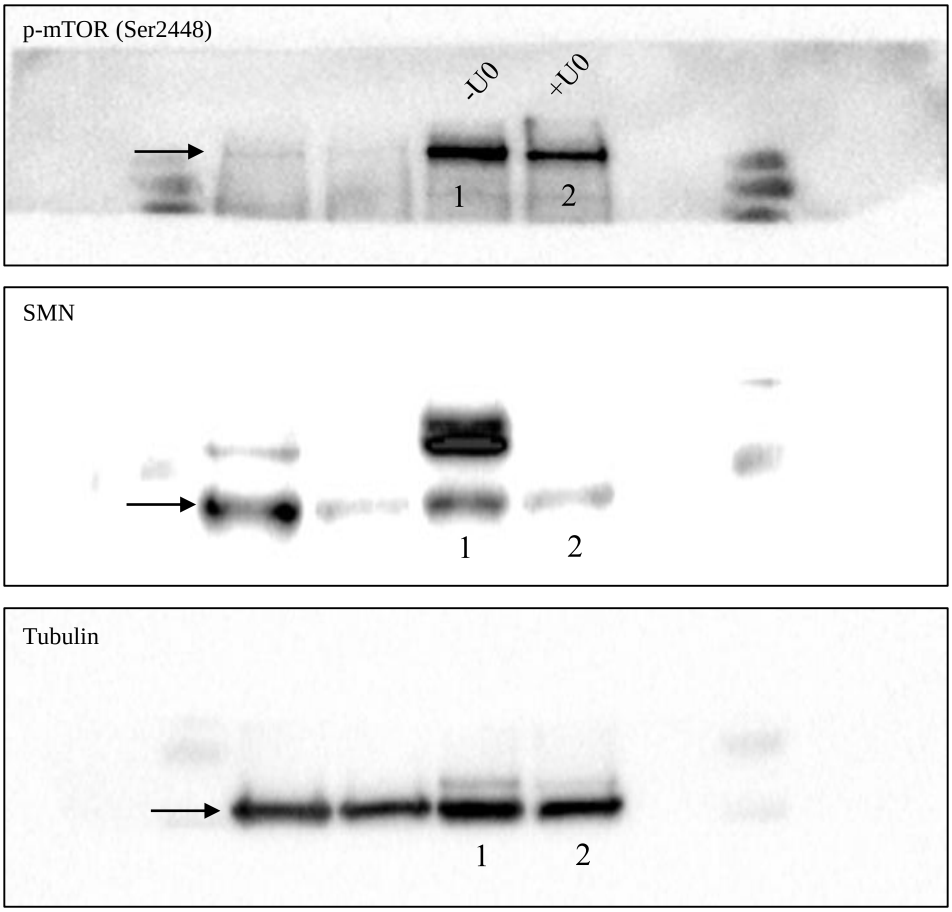

mTOR: Lanes 1, 2 are on the figure

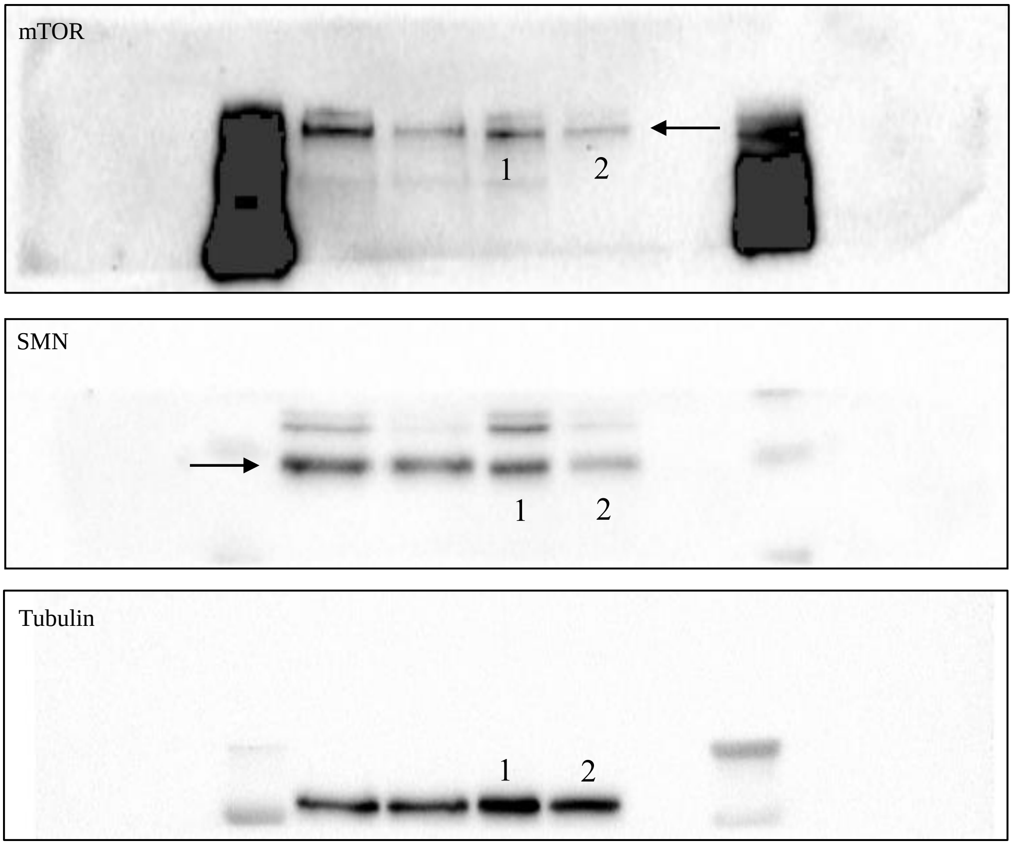

Full and uncropped western blot for Fig 4b

Left panel – p62 – Lanes 1, 2, 3, 4 are on the figure

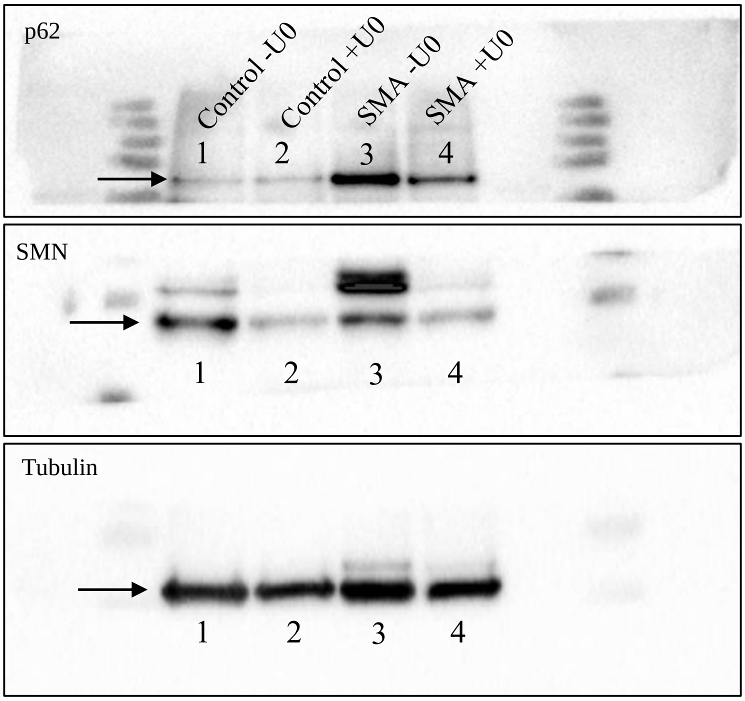

Right panel – LC3-II – Lanes 1, 2, 3, 4 are on the figure

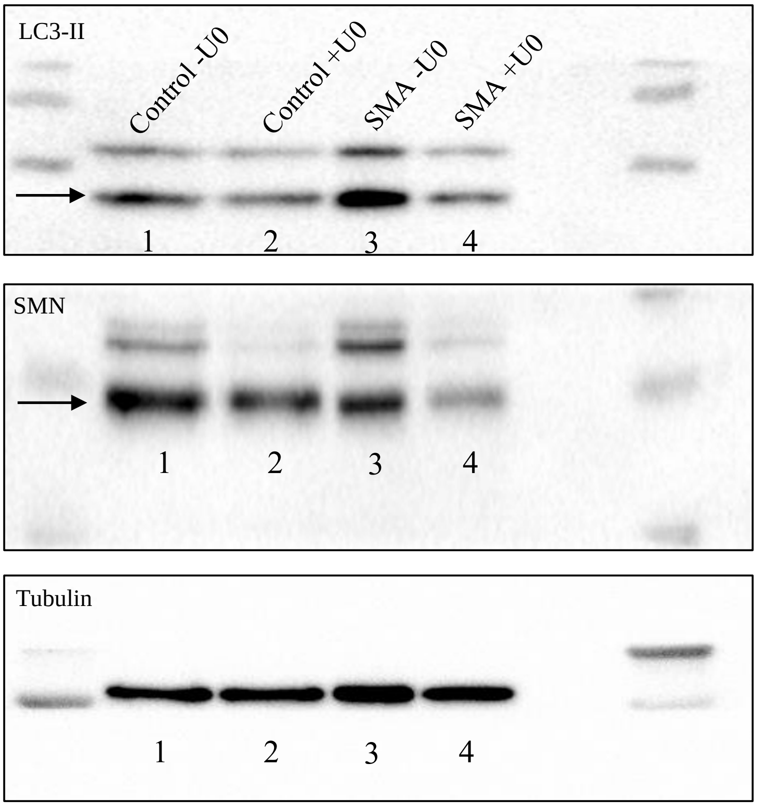

Full and uncropped western blot for Fig 5

Lanes 1, 2, 3, 4 are on the figure

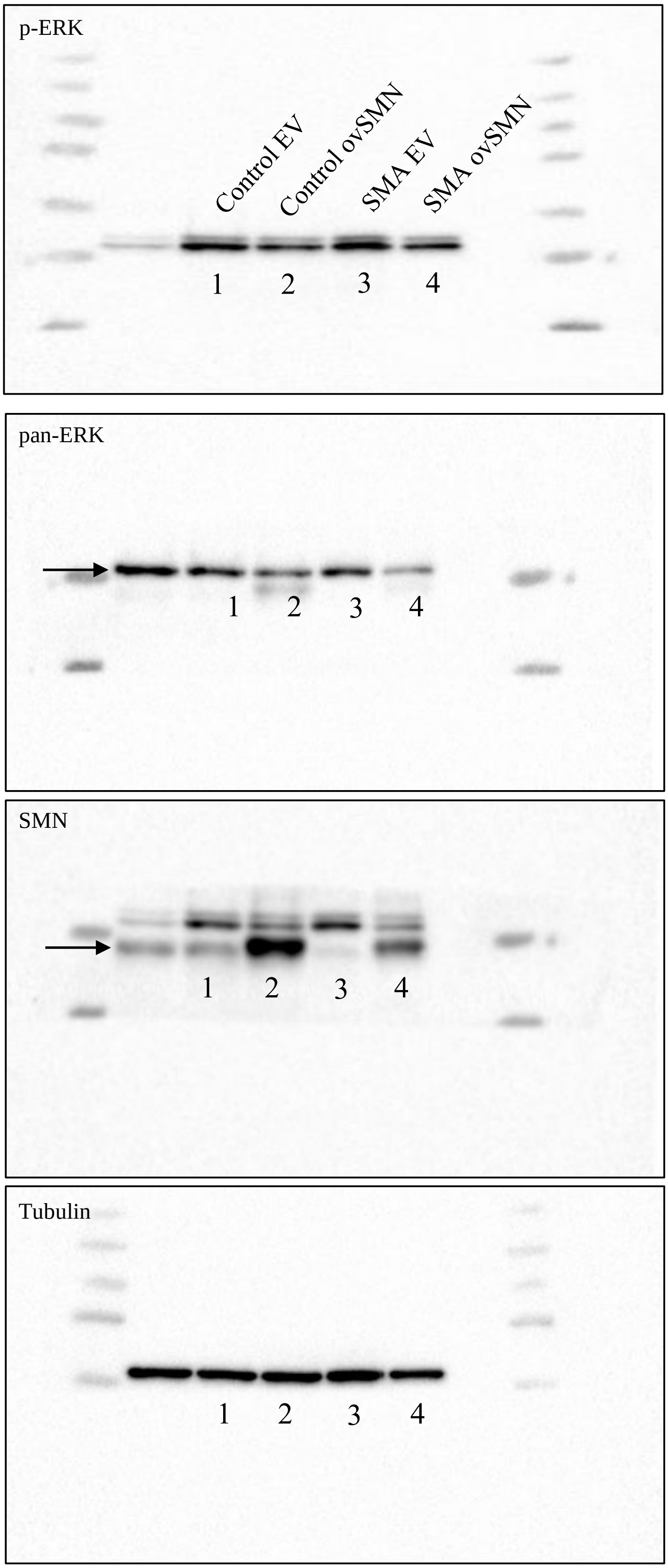

Full and uncropped western blot for Fig 6a

Lanes 1, 2, 3, 4 are on the figure

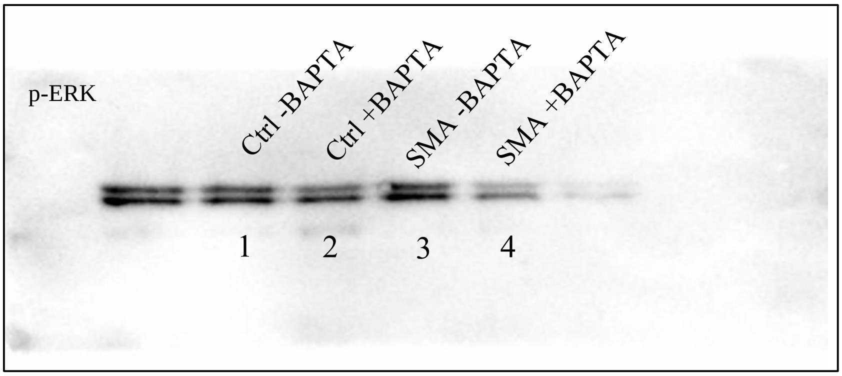

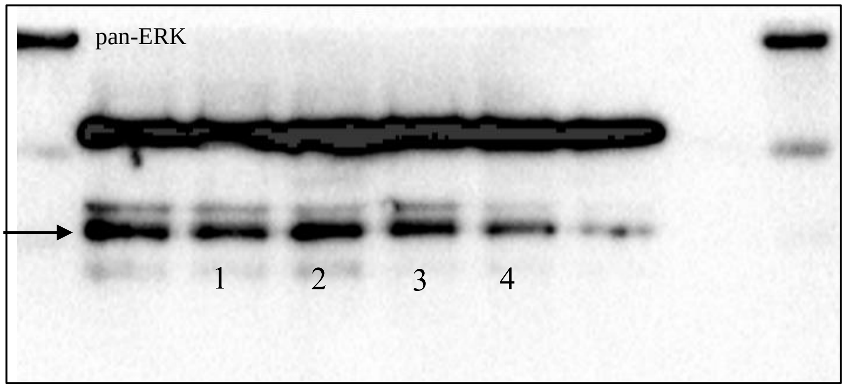

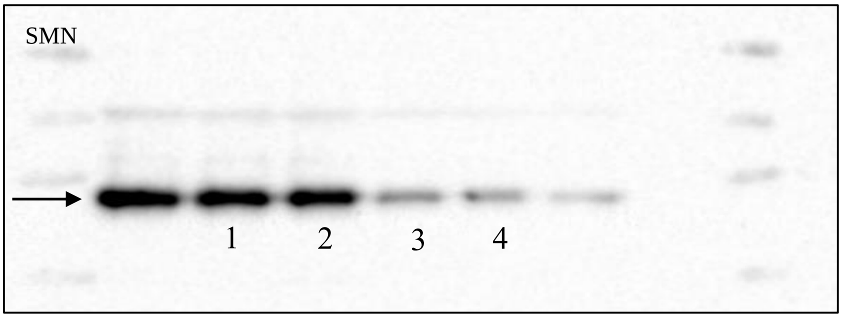

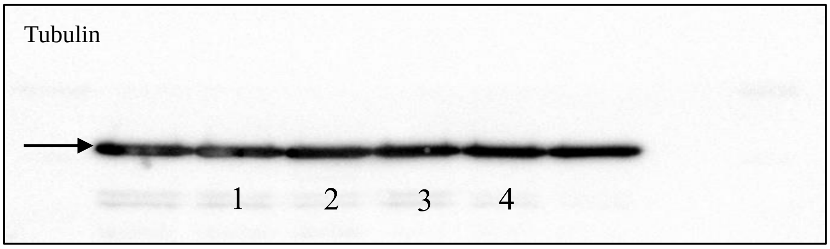

Full and uncropped western blot for Fig 6b

Lanes 1, 2, 3, 4 are on the figure

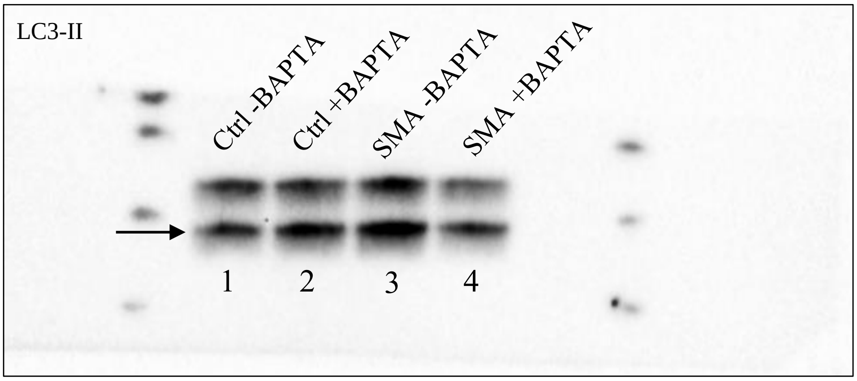

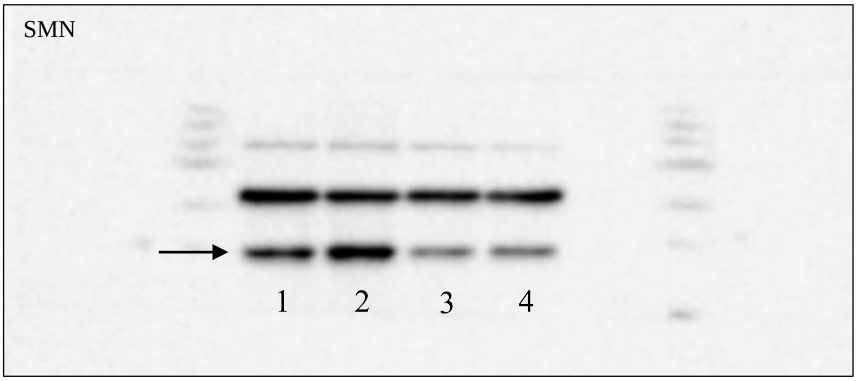

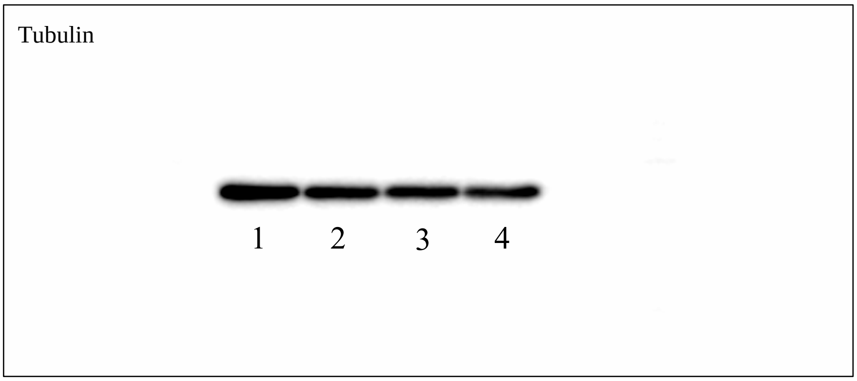

Supplement: Supplementary file 1 — Original western blot [file 41420_2023_1409_MOESM1_ESM.pdf]
